# Utilizing the Cyberforest live sound system with social media to remotely conduct woodland bird censuses in Central Japan

**DOI:** 10.1007/s13280-015-0708-y

**Published:** 2015-10-27

**Authors:** Kaoru Saito, Kazuhiko Nakamura, Mutsuyuki Ueta, Reiko Kurosawa, Akio Fujiwara, Hill Hiroki Kobayashi, Masaya Nakayama, Ayako Toko, Kazuyo Nagahama

**Affiliations:** Department of Natural Environmental Studies, Graduate School of Frontier Sciences, University of Tokyo, 5-1-5 Kashiwanoha, Kashiwa-shi, Chiba 277-8563 Japan; Center for Spatial Information Science, University of Tokyo, 5-1-5 Kashiwanoha, Kashiwa-shi, Chiba 277-8568 Japan; Japan Bird Research Association, 1-29-9 Sumiyoshi-cho, Fuchu City, Tokyo 183-0034 Japan; Fuji Iyashinomori Woodland Study Center, University of Tokyo, 341-2 Yamanaka, Yamanakako-mura, Minamitsuru-gun, Yamanashi 401-0501 Japan; Information Technology Center, University of Tokyo, 2-11-16, Yayoi, Bunkyo-ku, Tokyo Japan; Department of Regional Development Studies, Toyo University, 5-28-20, Hakusan, Bukyo-ku, Tokyo 1123-8606 Japan

**Keywords:** Live sound, Archive, Social media, Cyberforest, Audio census, Remote monitoring

## Abstract

We have developed a system that streams and archives live sound from remote areas across Japan via an unmanned automatic camera. The system was used to carry out pilot bird censuses in woodland; this allowed us to examine the use of live sound transmission and the role of social media as a mediator in remote scientific monitoring. The system has been streaming sounds 8 h per day for more than five years. We demonstrated that: (1) the transmission of live sound from a remote woodland could be used effectively to monitor birds in a remote location; (2) the simultaneous involvement of several participants via Internet Relay Chat to listen to live sound transmissions could enhance the accuracy of census data collection; and (3) interactions through Twitter allowed members of the public to engage or help with the remote monitoring of birds and experience inaccessible nature through the use of novel technologies.

**Electronic supplementary material:**

The online version of this article (doi:10.1007/s13280-015-0708-y) contains supplementary material, which is available to authorized users.

## Introduction

The spread of the Internet and mobile digital devices has given advent to a new scientific era, with scientists as well as members of the public accruing various benefits from rapidly evolving communication technologies. Parr (2008) stressed that shaping Internet technologies for ecology is an important focus in ecological informatics for the next century. This view is also borne out by researchers such as Ferster and Coops (2013), who recently examined the potential use of mobile personal communication devices for earth observation and suggested that scientists can view such devices as embedded sensors for environmental monitoring. Internet and mobile devices enable users to obtain various types of digitalized information from virtually anywhere on Earth, including remote locations, in real time. This realization motivated us to start our long-term Cyberforest Project^1^ under which systems for recording ecological information by unmanned automatic cameras and microphones in remote natural areas across Japan have subsequently been developed. Cyberforest is a large-scale information communication technology-based project that started in 1997 with the following primary goals: (1) to capture sound and imagery from forests in remote areas of Japan (to which thus few people have access) on a daily basis via unmanned camera and microphones over an extended period of time; and (2) to convert the data obtained into digital files that can be shared over the World Wide Web. Here we outline the basic system used in Cyberforest to collect such environmental data, and report on two experimental studies that test the systems’ ability to capture live sounds from a remote woodland expanse, plus how the subsequent interaction with social media can be used to conduct bird censuses remotely.

In earlier work, Saito et al. (2002) demonstrated that unattended recordings in a forest could be utilized to develop bird species lists; for this, they used a waterproof microphone and an unmanned automatic camera which collectively recorded 10 min of multimedia (video with audio) every day from November 7, 2000 to July 31, 2001. Subsequently, Saito and Shimura (2006) reported on the remote digital recording of bird song for 24 h (on June 2, 2004) in the same secluded forest, which allowed for the identification of a substantial number of bird species. The systems used in those two early experiments form the basis of our new system developed and reported on here.

In addition, several studies have begun to demonstrate the roles played by social media in the natural science field. Social media allows the rapid spread of information among individuals, a quality that holds the potential to promote participation, social learning, and ultimately alter public opinion (Büscher 2014). This theoretical potential is realized in a growing number of studies that show how social media can be used as effective tool for collaborative monitoring and information sharing in environmental research. For example, Daume et al. (2014) showed how long-term and large-scale forest monitoring approaches can be complemented by social media mining; and Joly et al. (2014) constructed an image-based plant identification tool for a social network of novice, amateur, and expert botanists, available as both a web and a mobile application. We explore these characteristic of participation in our own study and examine how live sound transmissions of birds on social media can be used for conducting bird censuses.

In this paper, we (1) outline the architecture of the Cyberforest live sound streaming system with its unmanned automatic camera and sound-recording components, (2) determine the utility of live sound recordings for conducting a bird census, and (3) examine the potential role of social media as a platform to disseminate and share such ecological live sounds between academics and non-academics. Finally, we discuss the significance and challenges associated with using live sound and social media for scientific monitoring.

## Development of the live sound system

### Study site

**Fig. 1:**
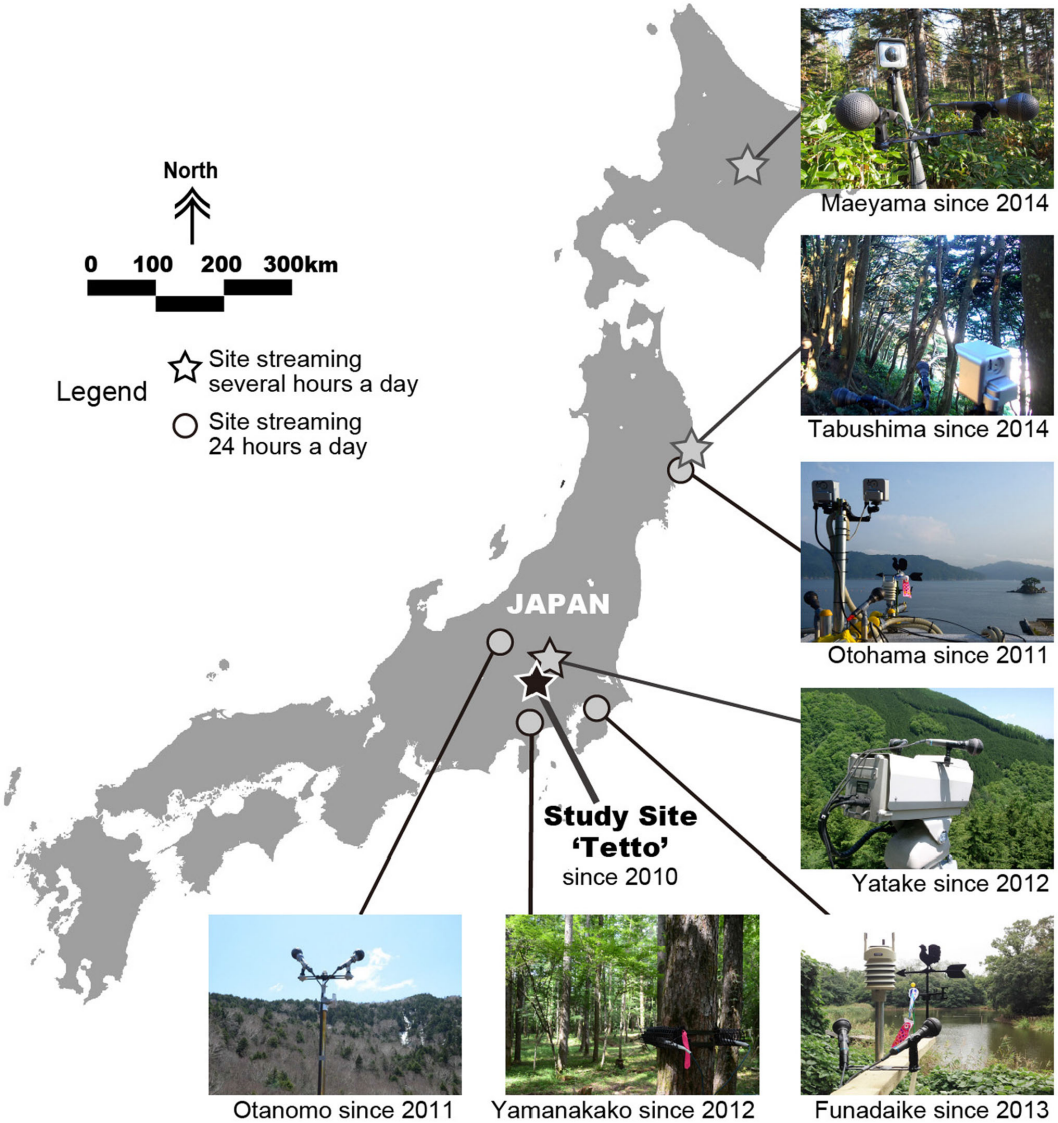
The location of the focal study site ‘Tetto’ used for live sound streaming, alongside those of the seven other remote ‘Cyberforest’ live sound streaming sites in Japan. The study site ‘Tetto’ is located at the University of Tokyo Chichibu Forest (Chichibu city, Saitama Prefecture, central Japan), and has live sound streaming operation since 2010. Locations of the other seven sites are: ‘Otohama’—International Coastal Research Centre, the University of Toyo (Otsuchi Town, Iwate Prefecture); ‘Otanomo’—Institute of Nature Education, Shinshu University (Yamanouchi Town, Nagano Prefecture); ‘Yatake’—University of Tokyo Chichibu Forest (Chichibu City, Saitama Prefecture); ‘Yamanakako’—University of Tokyo Fuji Forest (Yamanakako Town, Yamanashi Prefecture); ‘Funadaike’—Natural History Museum and Institute, Chiba (Chiba City, Chiba Prefecture); ‘Tabushima’—Tabuno Oshima Island (Yamada Town, Iwate Prefecture);‘Maeyama’—University of Tokyo Hokkaido Forest (Furano City, Hokkaido Prefecture)

**Fig. 2:**
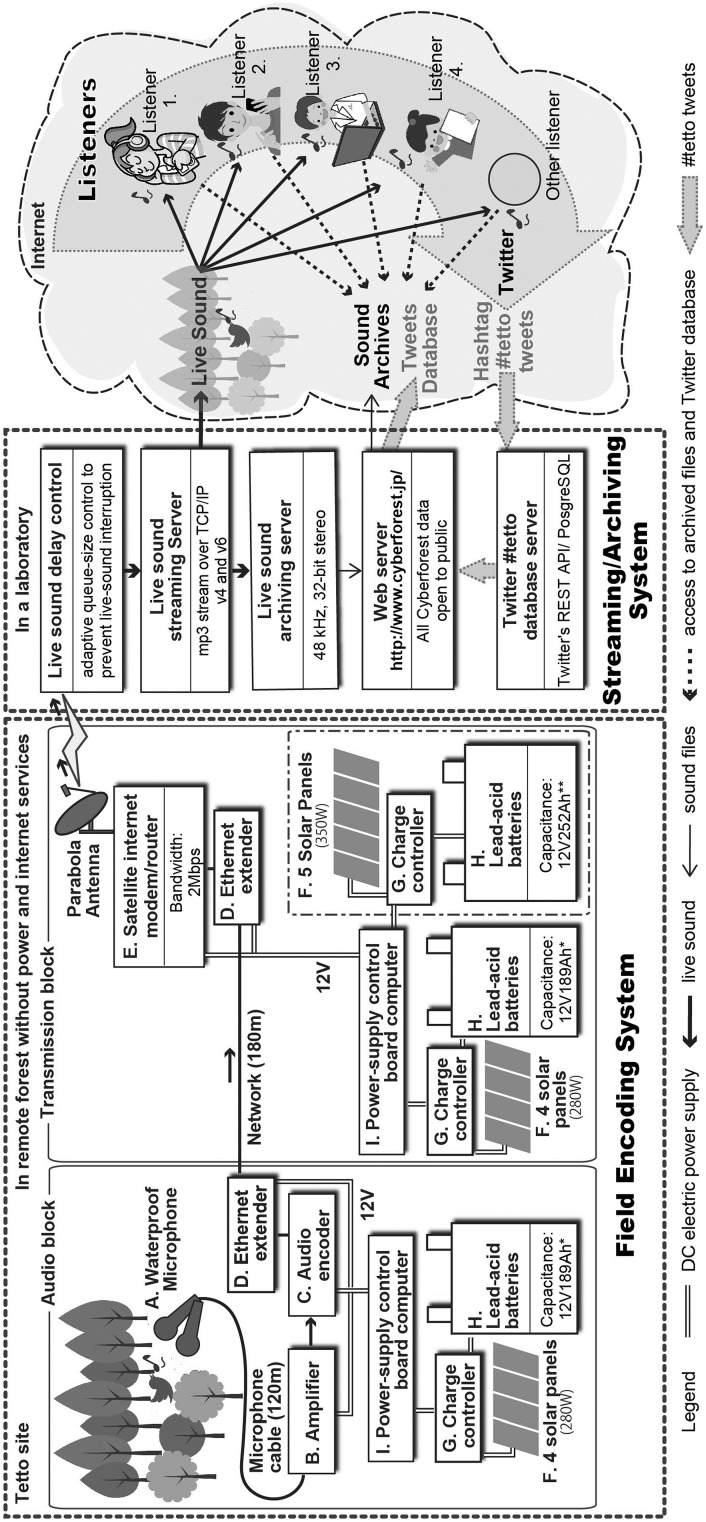
Block diagram of the Live Sound System since 2010. The Cyberforest Live Sound System consists of a Field Encoding System and a Streaming/Archiving System that began operating in March 2010. The Field Encoding System encodes the sound picked up by the microphones at Tetto forest in real-time, and transmits it to the Streaming/Archiving System, which is in the laboratory (University of Tokyo), via a satellite. The latter system controls the delay of the streaming from Tetto to prevent interruption of sound delivery through the Internet. The system also archives the recorded live sounds. Moreover, the Twitter #tetto Database Server collects tweets containing #tetto from Twitter and stores those in the database. Therefore, users can listen to both live sound and archived sound files and simultaneously access the collated commentary. Specifications: *A* Sony F-115; *B* PreSonus FireStudio Mobile; *C* Barix Instreamer-100; *D* Vigitron Vi2301; *E* IPstar network modem; *F* Sharp NE-70AIT (70 W); *G* MorningStar PS-30 M; *H* G&Y SMS27MS-730 (105 Ah); *I* EtherTexCircuits RMS-200

Figure 1 shows the location of the focal Cyberforest Project site ‘Tetto’ (35.9367°N, 138.8039°E). We used this site because satellite Internet was installed here first, back in February 2010 (Fig. 2). Tetto is part of a long-term ecosystem monitoring research site and located in the University of Tokyo’s Chichibu Forest,^2^ a remote area in Saitama Prefecture, central Japan. This research site comprises seven hectares of natural broadleaf forest, primarily consisting of two *Fagus* species intermixed with various conifer species. In addition, seven more sites across Japan were equipped for conducting live sound streaming as of November 2014 (Fig. 1).

### Cyberforest unmanned automatic camera system

**Fig. 3:**
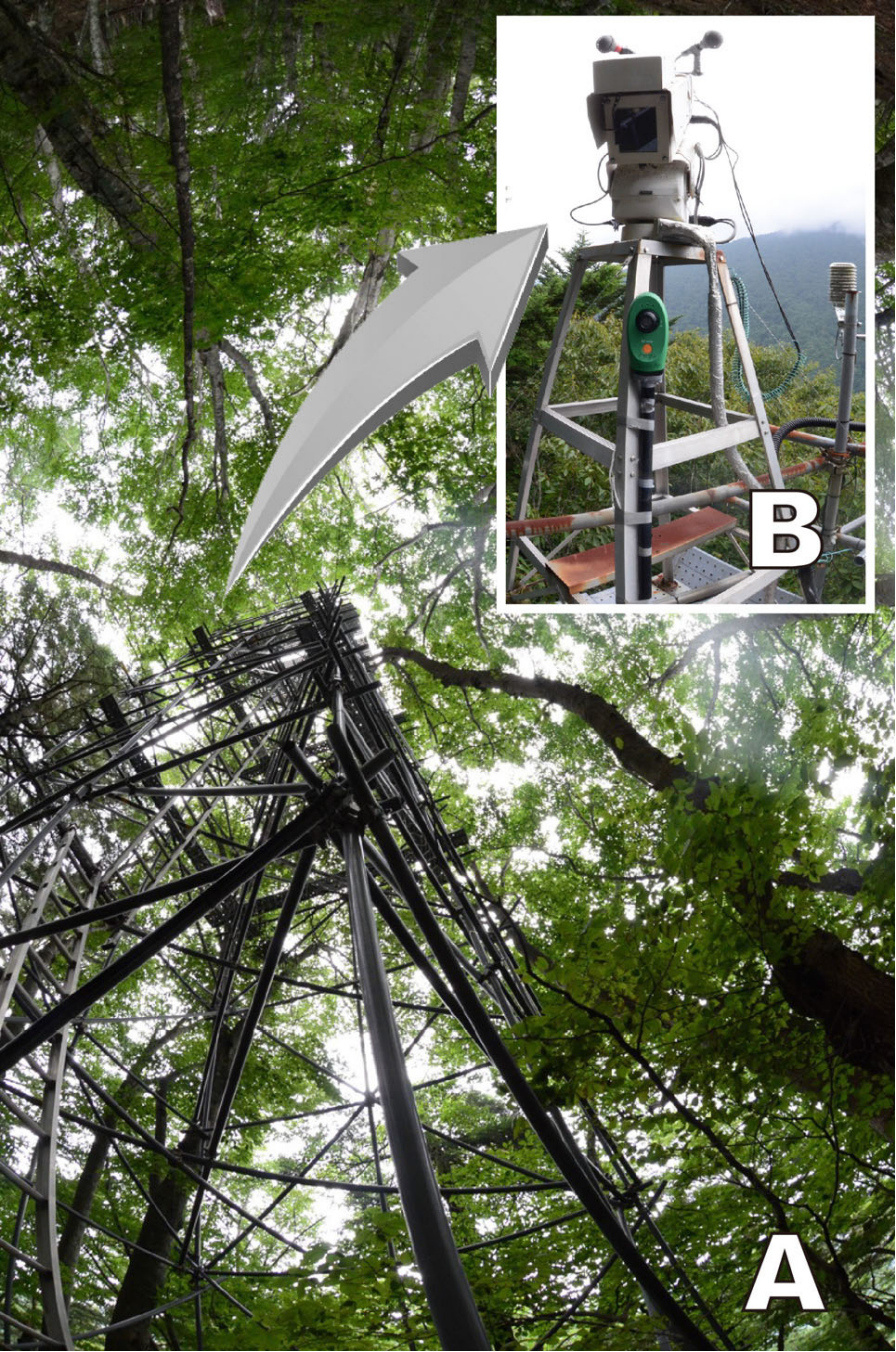
Unmanned automatic camera system at ‘Tetto,’ Chichibu Forest, central Japan, before connecting to the Internet (1996–2010). *A* Tree crown observation tower (23 m high) located among the beech trees of the natural forest. *B* Unmanned automatic camera installed at the top of the tower

We had previously developed and implemented the unmanned automatic camera system in 1996 for the Tetto site (Saito et al. 1998). This unmanned camera system was installed at the top part of an ecological observation tower built at a height of 23 m to observe the tree crown stratum (Fig. 3). Electric power to this system was supplied via a dynamo-electric generator, which was in operation between 11:30 am and 12:15 pm everyday via a timer-controlled starter. The camera took 40 video shots (each shot comprising a 15-s video) during the 45-min period, at various angles using the pan, tilt, and zoom functions programmed via a computer. We visited the Tetto site every 2 weeks to conduct maintenance work including changing the videotapes. The videotapes collected were digitized and edited at our laboratory, and made available for public viewing on the Cyberforest website (Fujiwara and Saito 1998). We found that three forest ecologists from the University of Tokyo and the Utsunomiya University in Japan had an interest in watching those videos as a sequential observation of phenology; yet, the absence of audio rendered it unsuitable for the general public. To broaden interest in the material produced, we installed a stereo microphone to record the natural sounds of the forest in March 2000. The resulting videos with bird songs and natural sounds were turned into teaching material that captured seasonal woodland changes (Saito et al. 2004, 2006). The materials were used between 2008 and 2011, at various schools in central Japan (five elementary schools (sixth grade) and one high school).

### Developing the Cyberforest live sound system

Working from the Cyberforest structure described above, we subsequently developed a ‘Live Sound System’ that enabled us (1) to listen to and record the sounds in a remote forest via an automatic camera system, and (2) to distribute those sound data to the public via the Internet, so that anyone could listen to the live sounds in the remote forest in real-time. The Live Sound System, depicted in Fig. 2, comprised two separate subsystems: a ‘Field Encoding System’ that digitized the live sounds in the forests; and a ‘Streaming/Archiving System’ that carried out live sound delivery via the Internet and archived the sound data in a recorded file. The technical operational testing notes of the Live Sound System can be found in Supplementary material 1.

#### Field Encoding System

The Field Encoding System (Fig. 2) established in the remote forest included an antenna and solar panel system (depicted in Supplementary material 2), thus capitalizing on the live distribution capabilities of the existing unmanned recording camera system. The Field Encoding System comprised two blocks: an audio block and a transmission block. The microphones of the existing unmanned cameras were individually connected to the amplifier of the audio block, and their outputs served as input to the audio encoder to convert microphone sounds into MP3, the format used for subsequent digital sound delivery. Because there was no prior Internet connection at the Tetto site, we incorporated a satellite Internet service which was provided by IPSTAR in February 2010. We subscribed to IPSTAR’s ‘Dual’ service plan, which served the largest upload bandwidth of 2 Megabits per second for approximately US $125 a month^3^; there was also a 2 Gigabyte daily limit on data usage per customer. Such characteristics of the service plan were important because research funds required to run a long-term ecological study are likely to fluctuate over time.

Electrical power to the Field Encoding System was provided by lead-acid batteries charged by a solar cell. Constraints on the power generation capacity meant that continuous operation of the Field Encoding System was not possible, and a control board-computer with a relay switch was incorporated into the power supply to facilitate control of its running time. The running time schedule was controlled by Cron software (a time-based job scheduler run on Linux). It was thus possible to control relay switches connected to the power supply of the Field Encoding System, and alter the operating time remotely if necessary. Four solar panels and two batteries were deployed for the audio and transmission blocks; the equipment used in these are listed A–I in Fig. 2.

We thought it was important for those listening to live transmissions to be able to experience changes in bird song within the day as well as across the seasons. Therefore, live sound was delivered year-round six times per day (with a total delivery time of 7 h and 50 min per day—see Table S1) from 2011 onwards. The longest transmission time each day was during sunrise (03:30–08:00 Japan Standard Time), which was when most bird song could be heard. Transmission also occurred around noon (11:00–13:00) to maintain consistency with the unmanned camera that had been used previously. Further 20-min transmissions occurred at 00:00, 02:00, 16:00, and 22:00 to capture the sounds of the forest and bird song during the evening and at night.

#### Streaming/Archiving System

The Streaming/Archiving System was located in the server room in our laboratory and had a normal bandwidth Internet connection, which allowed simultaneous public access to transmissions (Fig. 2). Two servers were used, one for streaming and the other for archiving. The servers were established in the laboratory because of the technical difficulties (e.g., previously mentioned problems with power supply or data download limits) involved in setting up and operating such a server at the relevant site. The processed audio signal was sent from the microphone, encoded into an MP3 live stream at the Field Encoding System, and transferred to the Streaming/Archiving System. The MP3 live stream could then be played simultaneously on MP3-based audio software around the world. We used a standard single package of Linux Fedora 14^4^ as an operating system and ICECAST2 server software^5^ for sound delivery. We have been using the ICECAST2 in our project from the beginning, and experienced only minor problems e.g., a slight delay (0.5 s) in transmission of sounds, but not anything that would affect user experience. However, signal strength in our system was affected by severe weather, such as heavy rain or a snowstorm, and this could cause a communication delay with the satellite network. To address this we adjusted the amount of information that could be received by the network (through the Linux TCP Receive Window) to ensure that interruption did not occur when transmitting sounds on the streaming server. Finally, in order to share the encoded live sound with listeners at any point or place the archiving server stored all the MP3 sound format files that were sent from the Field Encoding System through the Streaming Server.

## Testing the live sound system with social media to remotely census birds

We hypothesized that the Live Sound System could be used to conduct bird censuses by offering several ornithologists in different places simultaneous access, through the Internet, to live bird song recordings from a remote area. Communication between those ornithologists listening to the live bird sounds was made possible through the use of social media. Because both the live sounds and social media were openly accessible on the Internet, our setup also allowed members of the public to take part in a bird census. Consequently, we were able to examine the potential role of social media as a platform to disseminate and share live ecological recordings between academics and non-academics.

Two social media platforms were used to conduct the experiments, Internet Relay Chat and Twitter. Internet Relay Chat is an Internet application that facilitates transfer of messages in the form of text and is mainly designed for closed member group communication in discussion forums called channels (Oikarine and Reed 1993). There are many Internet Relay Chat server services, and numbers of users tend to be low, which helps to reduce delays in the delivery of messages. To conduct the first bird census study using live sound transmissions we opened a channel called #birdresearch on a free Internet Relay Chat server.^6^ We used Internet Relay Chat because log files containing species names, time and date information, and other identifiers, such as assumed behavior of birds and other animals, could be easily created. Twitter is a social media platform that enables users to send and read short messages called ‘tweets.’ In order to post tweets, an individual must have an account, which other users can follow to see their messages. The ‘retweet’ function on Twitter allows users to repost another individual’s tweet; this function means that a single tweet can sometimes reach thousands of users. Tweets can also be grouped according to a topic using the hashtag function (#), whereby users can search for all tweets created under the associated hashtag. For the purposes of this experiment, we used the hashtag #tetto to track tweets associated to the live sound transmissions.

### Experiment 1: Remote bird census on live sound using Internet Relay Chat

Standardized bird censuses conventionally use not only auditory but also visual cues because humans rely more heavily on such visual information. However, birds are highly vocal organisms, and approximately half of all bird species are Passerines, i.e., song birds that regularly communicate via vocalization. Therefore, auditory cues can play a major role in bird censusing, especially in dense forests where visibility is very limited.

The remotely conducted census developed by us depends exclusively on auditory sense, and we call it an audio census for short. We used the Live Sound System as the source of the auditory information and distributed this via the Internet on a real-time basis. Because only one set of microphones was installed in this system, it was not suitable for standardized censuses requiring multiple sample points. Therefore, we focused on collecting data across the breeding season in an effort to monitor changes in the phenology of forest birds.

The earlier studies of Saito et al. (2002) and Saito and Shimura (2006) focused at previously recorded sounds of the natural environment for short time periods (i.e., one day or less). These studies provided the basis for the establishment of a method for long-term monitoring of bird species through the use of a remote sound recording system. Here, we report the first case in which the remote sound delivery system was used to monitor vocal activities of birds across the breeding season, thereby aiming to establish this as a new ornithological survey method. The audio census allowed us to compile a list of the bird species at the forest site, using frequency of vocalization and other bird sounds (such as drumming of woodpeckers and wing beating of pheasants) to determine relative abundances. The utility of this newly developed audio census was determined by comparing its results with that of a conventional standardized field census conducted in parallel.

### Methods

As the platform for data recording and sharing, we used the #birdresearch channel on an Internet Relay Chat server, where three of the authors (including two ornithologists) accessed and posted the names and other ecological information of bird species and weather conditions while listening to the live sounds (Supplementary material 3). Each text posted on the Internet Relay Chat comprised a log file with a date and time stamp. We collected data on the forest avifauna for 70 min each day, starting 10 min before sunrise, between April 1 and June 30, for the years 2011–2013. We remotely listened to birds at the same site every day in 2011, except for eight days when the system temporarily failed because of snow covering the antenna. During the period 2012–2013, the number of Cyberforest study sites increased to three (see also Fig. 1); we monitored these alternately, thus listening to the Tetto site once every three days in those two years. Throughout a session, we recorded the bird species that vocalized every minute, so that we could calculate a species’ relative prevalence based on vocalization rate (Supplementary material 4; vocalization rate (%) = total minutes a species vocalized/total minutes all species vocalized × 100; vocalization included drumming and wing beating). We used vocalization rate of a species in a given year, and subsequently took the average of the three years.

In Japan, transect-based censuses traditionally played a major role in the collection of data on bird monitoring (Yui 1980). However, in 2008, spot census methodology was introduced in Japan because it causes less logistical problems and facilitates control over the study time period (Ueta et al. 2014). When we compared the results of the transect census with those of the spot census for each species, we found there was a strong correlation between the two types of censuses when the maximum number of individual birds at all the locations of the spot census was used (Hirano et al. 2009). Two spot census locations with a radius of 50 m were established at a distance of approximately 250 m from the microphones of the Live Sound System where the audio census information was collected. We visited the spot census locations 4 times in each breeding season between mid-May and mid-June from 2011 to 2013 and counted birds using both visual and auditory senses for 10 min within 2 h of sunrise. We used the maximum number of birds for the four visits to each spot in a year and took the sum of the two locations. The relative abundance of a species was calculated based on these annual values (relative abundance score of a species = annual total of a species/annual total of all species × 100). We took the average of the relative abundance scores of three years and compared these with audio-census data.

### Findings

We listened to the live sounds through the Internet and shared the species of birds identified, as well as other activities observed at the site, by writing a text into the #birdresearch channel chat facility. Two ornithologists posted the species names corresponding to the songs and calls they heard in the live sound minute-by-minute to the Internet Relay Chat channel #birdresearch. Despite minor problems with time lags in transmissions (1–10 s), communication over this channel proved helpful for species identification. For example, when the song of a Red-billed leiothrix (*Leiothrix lutea*), an introduced species which occurs sporadically in more southern regions of Japan, was heard, a birder who had joined the Relay Chat channel identified it because he was more familiar with it. Furthermore, archived files were played when it was necessary to listen to certain sounds again, for example to correct mistakes or determine species that could not be identified during the live delivery period (Ueta et al. 2012).

**Fig. 4:**
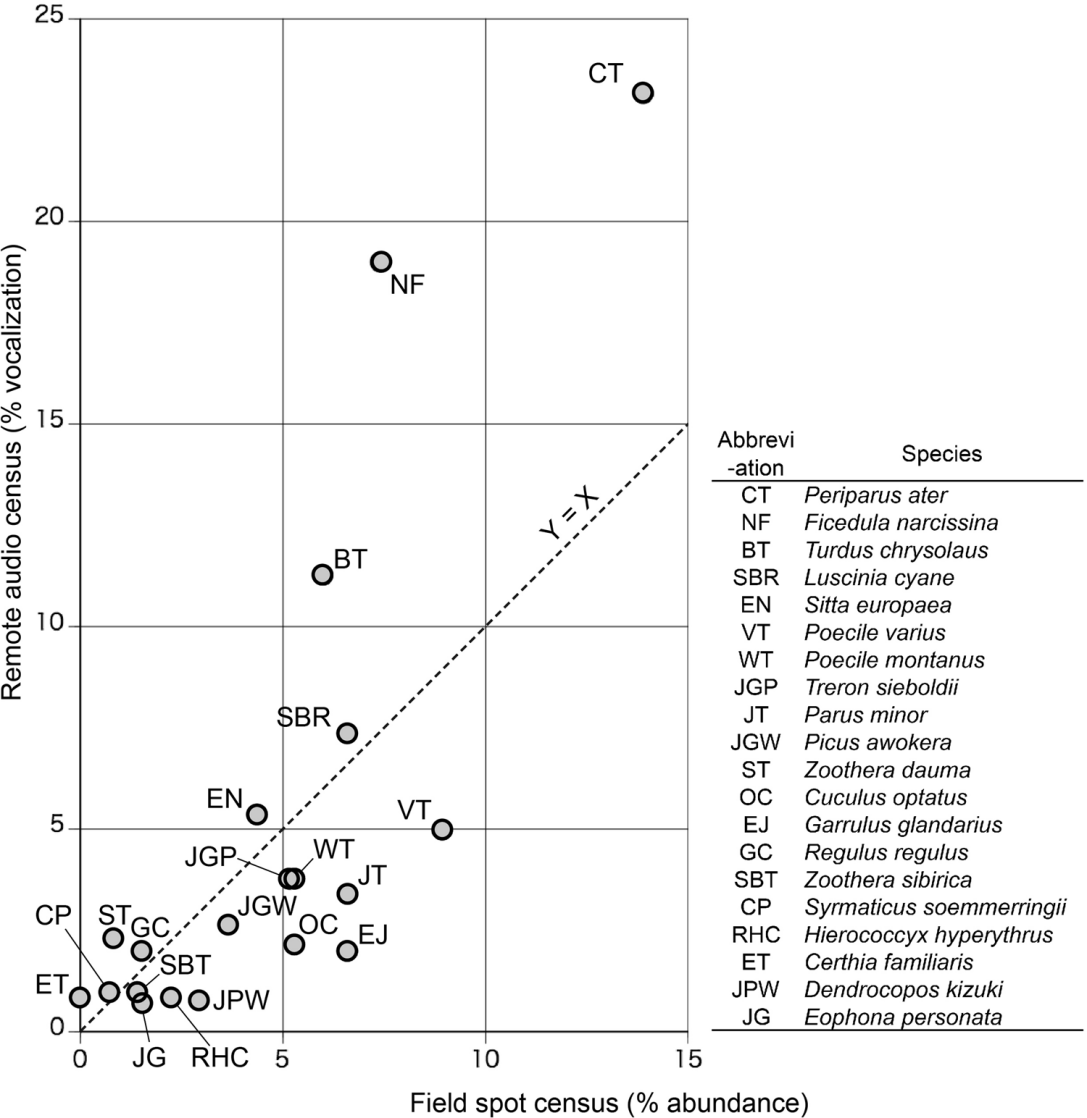
Relationship between the relative abundance of the 20 most commonly observed bird species determined through remote audio census (% of all vocalizations) and through field-based spot census methodology

A total of 36 bird species were recorded in the audio census over the 3-year study period, while carrying out the field census a total of 28 species were detected during the same period (Supplementary material 5). All but two species detected during the field census were also recorded and subsequently identified through the audio census. The two species that were not recorded in the audio census were a rare species and a large woodpecker that was difficult to identify to the species level by sound. The 20 most abundant species in the audio census accounted for 90 % of species recorded in the field census. Figure 4 shows the correlation between recordings of these 20 species in the audio census and the field spot census (Kendall tau rank correlation coefficient *τ* = 0.576, *n* = 20, *P* < 0.001). Species for which very similar values were obtained with either method (i.e., those nearest to the dotted diagonal in Fig. 4) were Siberian blue robin (SBR), Eurasian nuthatch (EN), goldcrest (GC), and Japanese grosbeak (JG). Also somewhat similar values were obtained for Japanese green pigeon (JGP), willow tit (WT) and Japanese pygmy woodpecker (JPW).

The species that appear to the left of the diagonal line in Fig. 4 were over-represented in the audio census. These were coal tit (CT; ranked highest for both), narcissus flycatcher (NF), brown-headed thrush (BT), and copper pheasant (CP). Both coal tit and narcissus flycatcher are abundant but also very vocal species. The difference in recordings of the brown-headed thrush is likely due to the difference in time of the day when audio and field censuses were taken, as this species is most vocal around sunrise. Copper pheasants are usually extremely difficult to detect because they are ground-dwelling and avoid people. It was assumed that they made displays near the unmanned microphone system because they were not disturbed by human presence.

Species on the right of the diagonal line are under-represented in the audio census. These were varied tit (VT), Japanese tit (JT), and Eurasian jay (EJ). Varied and Japanese tits, which are not particularly vocal, were more easily detected in the field census when also using visual cues. Eurasian jays, which typically move around in small flocks, were consistently recorded as one bird in the audio census, thus leading to underestimation. The drumming sound of copper pheasants was around 300 Hz or lower, which fell at the lowest end of the sound-frequency spectrum of target birds in this study. It is high enough for humans to hear in the field, because our auditory range is approximately 20–15 000 Hz. However, if the field conditions are unfavorable for hearing, such as when the site is noisy, the target bird is far away, or a researcher is not used to the drumming sound, it can go unnoticed. The regular built-in speakers of a personal computer are not designed to reproduce this low-frequency sound. When listening to the live sounds of Cyberforest for bird monitoring, therefore, the use of ear-phones or head-phones are recommended because they can reproduce the low-frequency sound.

### Experiment 2: Harnessing wider interest in bird song and censusing using Twitter

In our second experiment, we wanted to determine whether we could draw in listeners through Twitter by announcing that bird songs in distant natural forests were available to listen to in real-time, provide them with tweets giving information on each of the vocalizations, and encourage them to take part in the bird censusing process.

### Methods

First, we implemented a database in the Streaming/Archiving System to collect tweets. This was done by archiving any tweets that used the #tetto hashtag and storing them on our ‘Twitter #tetto Database Server’ in PostgreSQL database^7^ format (Fig. 2), which is open to the public.^8^ This database, in which tweets were stored with their date and time stamp, enabled us to search for the corresponding sound files in our archiving server using date and time as retrieval keys. We recruited listeners through Twitter by announcing the live sound, and explaining how to listen to the live recordings, from April 16, 2011 to December 16, 2012 (the number of recruiting tweets in 2011 and 2012 were 636 and 219, respectively). At that time, we asked listeners to use the hashtag #tetto to indicate the origin of the live sounds when tweeting, which simultaneously facilitated the sharing of the live sound listening experience with other listeners. The construction of this database also allowed us to examine whether collected #tetto tweets could be used as metadata, enabling the live sounds to be searched according to a date and time key.

### Findings

**Fig. 5:**
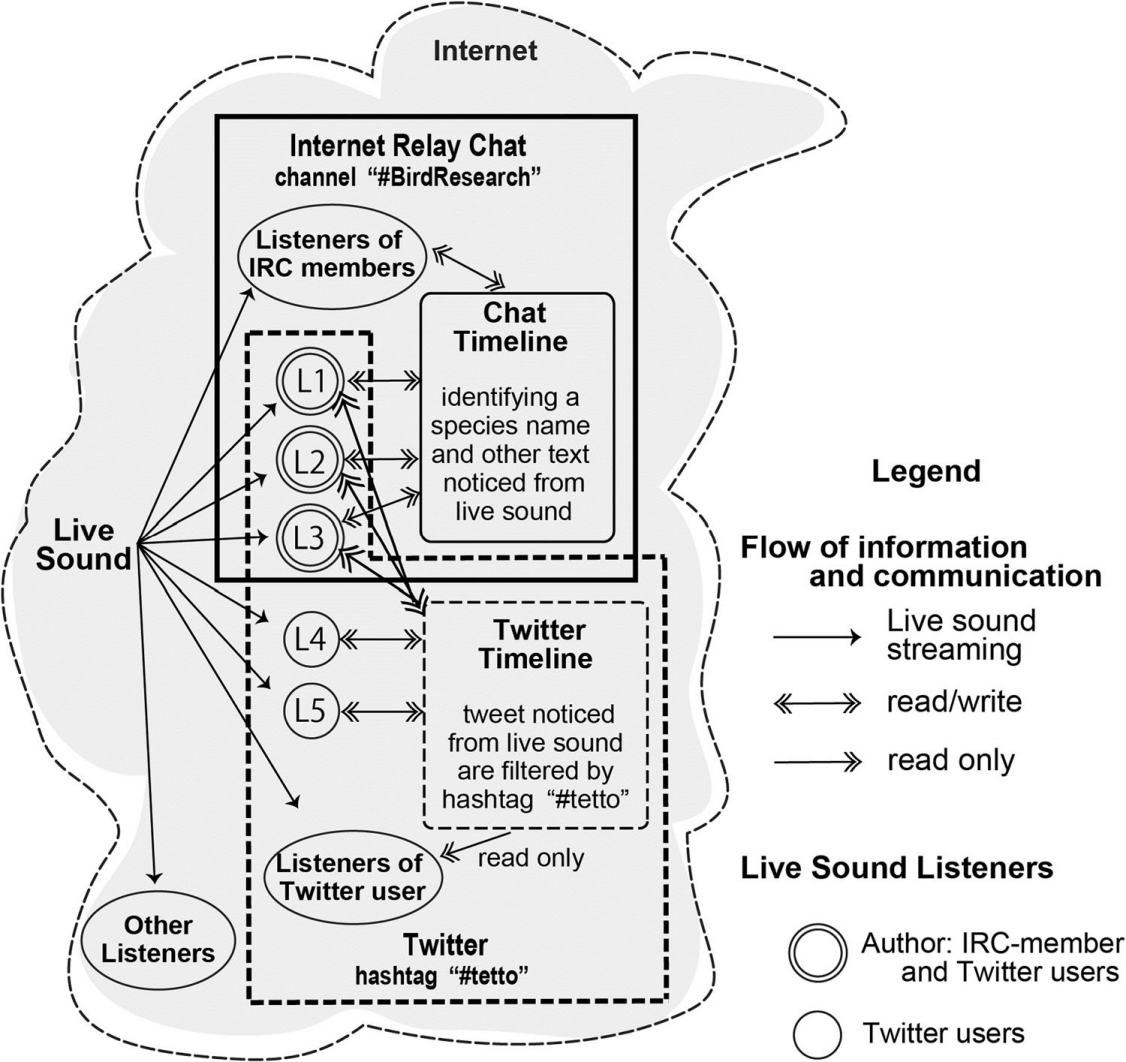
Block diagram of Live Sound Listeners and Social Media (Internet Relay Chat (IRC) and Twitter), with detail representing the situation on a specific day (April 21, 2011). In this case, the listeners to live sound on the Internet were IRC channel #birdresearch members, Twitter users, and other listeners. The IRC channel members identified the species of birds heard during each minute of live sound, posting it in the Chat timeline while also reading the Twitter timeline. L1, L2, and L3 were authors of this article, with L1 and L2 being ornithologists. L3 also tweeted announcements of the live sound service to recruit new listeners. Twitter users, who could listen to the live sound, were able to read the messages about the live sound by filtering on the hashtag #tetto. L4 was an ornithologist and L5 an unknown listener. Because the listeners that used Twitter have their own followers, their tweets or retweets about the sound transmissions spread widely through their own followers

When announcing the live sounds via Twitter, the streaming server was accessed more than 1462 times between January 6 and June 1, 2011. Also, 1912 tweets with the hashtag #tetto were recorded over 75 days from April 17, 2011 to June 30, 2011. The breakdown by user category was as follows: five authors posted a total of 1065 tweets, one ornithologist with extensive research experience at the study site posted 391 tweets, and another 115 tweeters were responsible for posting a further 456 tweets. Figure 5 and Supplementary material 6 show the case, on April 21, 2011 between 5:41 am and 6:07 am, in which one of us (L3 in Fig. 5) first announced the live sound on Twitter, leading to a listener (L5 in Fig. 5) gaining interest after seeing the tweets of #tetto, while simultaneously listening to the live sounds together. At the same time, two ornithologists (L1 and L2 in Fig. 5) identified species every minute while listening to the live sounds in the #birdresearch channel of the Internet Relay Chat, as in Supplementary material 6. The contents of the tweets, produced by ornithologists as well as listeners, included commentaries, questions, and explanations of the live sounds heard. In some cases, clear positive emotions were expressed such as by a listener, who commented that they felt as though she or he were part of a museum tour with the personalized commentary of a curator.

There was also a case in which the number of concurrent listeners increased rapidly when unusual ecological sounds were heard in the field over a 10-min period from 6:59 am on February 21, 2012. In this case, corresponding tweets were posted for a 20-min period from the start of the sound; approximately 19 people were recorded listening to the sound delivery during that episode. The unknown sounds ceased without anyone determining what species had produced it or why. We subsequently sent the recorded file to the ornithologist described above, who identified it as the sound of a territorial dispute between white-backed woodpeckers (*Dendrocopos leucotos*). We later uploaded this identification to Twitter with a link to the audio recording posted on the sharing site SoundCloud,^9^ which was repeatedly uploaded and played thereafter (56 times in 2012, 25 times in 2013, and 54 times in 2014). As tweets with the hashtag #tetto were publically accessible from our database, Twitter users were able to share songs of birds together with commentary on them.

## Discussion

The Live Sound System demonstrates that it is possible to deliver ecological sounds of remote natural areas without readily available supplies of power or an installed online network. The system developed here delivered sounds year-round during specific times of the day (e.g., around sunrise, noon, and midnight), which allowed listeners to experience bird sound from our focal site since 2011 until today. As system managers and listeners could check the live sound almost every day, any system troubles could be discovered promptly and corrected. Indeed, system maintenance at the Tetto site was performed regularly, at least every two weeks, and we kept improving the Field Encoding System.

When ornithologists, based in separate places, used the developed sound system to census woodland birds remotely, more species were detected than during the field-based spot-censuses (36 vs. 28). The finding that the top 20 species in the audio census accounted for 90 % of those in the field census suggests that the Live Sound System has clear potential for conducting bird censuses. It captured well those bird species that were vocal, occurred in relatively large numbers, and produced sounds that were of a sufficiently high frequency for speakers to reproduce well. The successful recording of sounds of an extremely secretive and easily disturbed species suggests that it has also utility for monitoring bird species whose behavior is readily affected by people.

There were several differences in methodology employed between the audio census and the field census reported on in this paper. The audio census was carried out around sunrise because it is the time when most of the birds are active. We continued to collect data for 70 min including an hour after sunrise in order to also pick up the vocalization of species that become active in this time zone. The vocalization data were sampled for three continuous months to determine the many phases of bird activity throughout the breeding season. In the field census, on the other hand, the data were collected within 2 h after sunrise, sometimes after the audio census time. The spot census was conducted for 10 min per day and a total of 80 min in four plots per year. This amount of time is much smaller compared to that of the audio census (approximately 2000 min). These differences in methodology between the audio and spot censuses are difficult to evaluate, but likely play a part in the difference in prevalence values of some bird species between the two censuses.

We demonstrated that anyone could hear the live sounds of a remote forest wherever they are as long as there is an Internet connection. The Cyberforest Live Sound System also enables city dwellers, for example, to experience bird song in remote natural areas to which they may not otherwise have access. The Live Sound System made it possible for us to conduct the audio census every morning for three months for three consecutive years—something that would have been close to impossible otherwise, given the remoteness of the area and inclement weather conditions hindering physical access to the site. The commenting on the bird song and wider broadcasting was dependent on input by the researchers who simply enjoyed sharing the experience of listening to bird song and other vocalizations with other people. Our study showed that the integration of live sound and social media allowed listeners to learn from other listeners and engage with a remote area, potentially fostering a sense of closeness to some of Japans’ most remote natural places.

By incorporating tweets with Twitter hashtags concerning live sounds in a database, we were able to tag some of the (highly variable) characteristics of remote environments as metadata. For example, when a user searches for a Twitter log with the keyword “owl,” a list of places showing where the hooting of owls can be listened to, together with the date and time of the recording, is displayed. The user could then locate the corresponding section of the sound archive file to check. This means that someone, who has never heard the voice of an owl before could get to know the day and time of the owl’s call, and then try to hear it at that same time and place in real time. It is difficult to traverse a remote forest at night, but it is easy to give the Live Sound System a try.

Finally, applying the technologies developed, we have been expanding the live sound monitoring, as shown in Fig. 1, to sites 1 through 7.^10^ At site 6, an unmanned breeding island for streaked shearwater (*Calonectris leucomelas*), we started the live sound test operation experiments without maintenance using only the solar panel and battery system developed in October 2014 and, as of October 2015, it is still operating as intended.

## Conclusion and perspective

We have developed a new type of bird census method coined ‘audio census,’ using a Live Sound System and social media (Internet Relay Chat and Twitter). A total of 36 bird species were recorded by several ornithologists in separate places using the audio census method during the 3-months-long breeding seasons of the 3-year study period. We showed that the Live Sound System and social media could contribute to raising public interest in, and realizing auditory real-time experience of, remote natural forest. The Cyberforest Live Sound System is an unmanned automatic system that facilitates information collection in a remote forest without having to visit the site each time, creating a new way to conduct bird monitoring with fewer logistical problems for humans and less disturbance of wildlife. The use of social media enables a large number of participants, including professional and amateur ornithologists, to participate, thus building a base for a large-scale monitoring scheme with participants from all over the country. Expecting that the Cyberforest Live Sound System can contribute both to the monitoring of natural environments and engagement of the wider public in nature conservation, we consider it important to keep pace with the quickly developing digital communication era.

## Electronic supplementary material

Supplementary material 1 (PDF 298 kb)

## Footnotes

1 http://www.cyberforest.jp/.

2 http://www.uf.a.u-tokyo.ac.jp/english/our_forests/UTCF.html.

3 http://www.ipstar.com/jp/en/package.html.

4 http://fedoraproject.org/.

5 http://www.icecast.org/.

6 http://www.irc.friend-chat.jp/.

7 http://www.postgresql.org/.

8 http://cf4ee.nenv.k.u-tokyo.ac.jp/drupal7/twitLog/twitAnHour.

9 https://soundcloud.com/kaorusaito/20120221073331at26m10s-10m.

10 http://www.cyberforest.jp/.
