# Supplementary material for: Utilizing the Cyberforest live sound system with social media to remotely conduct woodland bird censuses in Central Japan
Source: Ambio. 2015 Oct 27;44(Suppl 4):572–83. doi: 10.1007/s13280-015-0708-y (PMC4623865; doi:10.1007/s13280-015-0708-y)
Supplement: Supplementary file 1 — Supplementary material 1 (PDF 298 kb) [file 13280_2015_708_MOESM1_ESM.pdf]

Ambio

Electronic supplementary material

Title: **Utilizing the Cyberforest live sound system with social media to remotely conduct woodland bird censuses in Central Japan**

Authors: Kaoru Saito, Kazuhiko Nakamura, Mutsuyuki Ueta, Reiko Kurosawa, Akio Fujiwara, Hill Hiroki Kobayashi, Masaya Nakayama, Ayako Toko, Kazuyo Nagahama

Supplementary material 1

Technical operational testing note of the Live Sound System

We verified the efficacy of the Live Sound System by checking its sound quality, control of distribution time, and the production of sound record files from live sound. In particular, we needed to ensure continuity and stability for several hours, at least in the early morning, which is the most important time frame for a bird census. The Streaming/Archiving System located in the laboratory was operational 24 hours per day, but intermittent operation was programmed so that the Field Encoding System had room to operate without solar power generation for a maximum of three days while using the battery charged by the solar cell as a power source. Generally, a deep-cycle battery is designed to discharge an average 60% of its capacity, and there is a direct correlation between the depth of discharge of the battery and the number of charge and discharge cycles it can perform (http://en.wikipedia.org/wiki/Deep_cycle_battery). We assumed the maximum percentage of discharge of the full battery capacity to be 50% because we considered a 10% safety margin to prevent a shortened battery life due to deep discharge. Furthermore, the fully charged batteries need to have sufficient excess capacity for three days without sunlight in remote area. Thus, a total available discharge capacity of 189 Ampere-hour (Ah) at 12Volts (hereinafter abbreviate ’at 12V’) as in Figure 2 would expend 31.5 Ah per day according to calculation. The operational testing process was as follows: we set the audio encoder’s three parameters (the channel mode, the encoding format, and the Mpeg Encoding quality) to mono, Mpeg-1 Audio Layer-3/ frequency 16kHz, and record leakage, respectively. Live delivery and pilot recording operation for a total of 140 minutes per day were started on March 9, 2010, and we

repeatedly checked the live sound delivery performance, power management accuracy, delivery sound quality, and recording file generation.

The power consumption by the audio block and the transmission block was approximately 1.5Ah and 3.5Ah, respectively. Delivery and recording release were conducted for a total of 410 minutes, 6 times per day. On March, 5, 2011 batteries and solar panels were added to the transmission block as illustrated in Figure 2. The power supply batteries were rotated on an on-going basis. Finally on May 12, 2012, we set the audio encoder's parameters as follows: channel mode, stereo; encoding format, Mpeg-1 Audio Layer-3/ frequency 32kHz; MPEG Encoding quality, 7 (the highest level). Further, we extended the total delivery and the recording time to 470 minutes as shown in the following Table S1. The power consumption was 12.3Ah by the audio block and 28.6Ah by the transmission block per day. The final audio parameters comprised a communication bandwidth of approximately 200kbps, and the recoding file size per an hour should have been 72MB per hour, using simple arithmetic. However, empirical operational tests showed that the actual size of the sound recording file for one hour in the server was 82MB because ICECAST2 saves as 48kHz 16bit stereo MP3 for a total 490 minutes; thus the total size of the recording files was approximately 0.6GB higher (Table S1), but still below the data limit restriction of 2GB. This total file data size, almost one-third of the data limit, for live sounds is appropriate also for other data communication, such as weather data and photos, via the satellite internet. The fully charged batteries have sufficient excess capacity for three days without sunlight, but require several days of uninterrupted sunshine to become fully charge. Finally, the system is operating smoothly without any power supply problems but there were

several times that a satellite internet was cut off because the parabola antenna got covered with snow during the study period.

Table S1 Live sound delivery time table and recorded sound file size

Daily delivery order number	Time table since May 12, 2012*		Recorded sound file** size (MB)
	Japan Standard Time	Coordinated Universal Time	
1st.	00:00 – 00:20	15:00 – 15:20	23
2nd.	02:00 – 02:20	17:00 – 17:20	23
3rd.	03:30 – 08:00	18:30 – 23:00	364
4th.	11:00 – 13:00	02:00 – 04:00	160
5th	16:00 – 16:20	07:00 – 07:20	24
6th.	22:00 – 22:20	13:00 – 13:20	22
Total	7hours 50minutes		616

* http://cyberforest.nenv.k.u-tokyo.ac.jp/tetto-sound/2012/

** MP3 (48kHz 32-bit stereo)

Supplementary material 2

Parabola antenna of the satellite Internet and solar panels at the Tetto site

The parabola antenna and solar panels were installed in an open area under the southern sky. Electricity was only generated efficiently three hours per day by the solar panels because they were surrounded by trees that towered over them to heights of up to 20m. Several days with continuous sunshine were needed to have the solar panels fully charge the batteries.

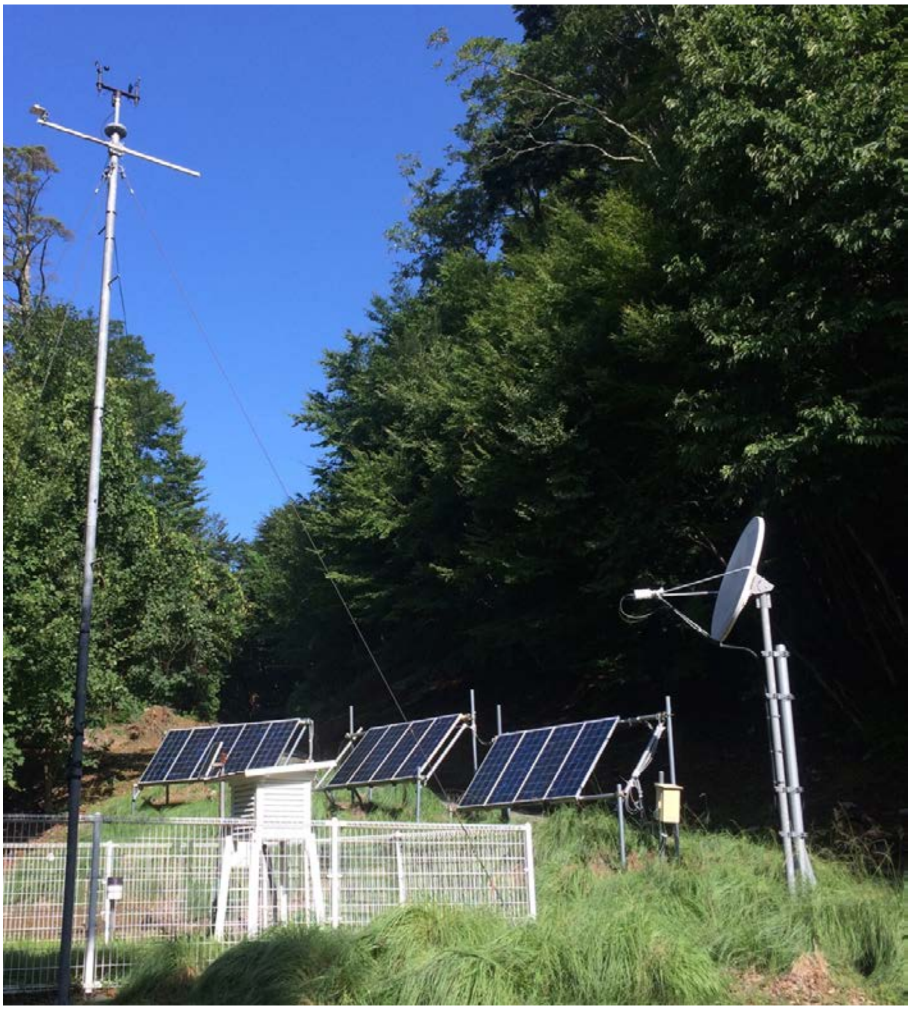

Supplementary material 3

An example of the chat log (May 7, 2014).

The channel called #birdresearch on a free Internet Relay Chat server was opened as a platform for participants in the audio census to note bird names and communicate with each other.

Time	Recorder	Species code	English name	Behavior*	Note
4:41	MJUeta	A	Coal tit	S	
4:41	MJUeta	B	Brown-headed thrush	S	
4:41	MJUeta	C	Willow tit	S	
4:41	MJUeta	D	Siberian blue robin	S	
4:41	MJUeta	E	Narcissus flycatcher	S	
4:41	MJUeta	F	Japanese green woodpecker	C	
4:41	MJUeta	G	Varied tit	S	
4:41	RKurosawa	H	Japanese green pigeon	S	"In the distance"
4:42	RKurosawa	I	Large picidae	D	
4:42	KSaito				"Good morning! Are we listening to the 'Tetto site' today?
4:42	MJUeta				"Good morning. Yes, we are."
4:42	MJUeta	A	Coal tit	S	
4:42	MJUeta	B	Brown-headed thrush	S	

4:42	MJUeta	C	Willow tit	S	
4:42	MJUeta	G	Varied tit	S	
4:42	MJUeta	D	Japanese green woodpecker	S	
4:42	MJUeta	F		C	
4:42	RKurosawa				“The drumming I recorded may have been the chopping of a helicopter.”
4:42	RKurosawa	J	Goldcrest	S	
4:43	MJUeta	K	Eurasian nuthatch	S	
4:43	MJUeta				“I think so too.”
4:43	MJUeta	A	Coal tit	S	
4:43	RKurosawa				“Then remove the Large picidae at 4:42 from the data.”
4:43	MJUeta	B	Brown-headed thrush	S	
4:43	MJUeta	C	Willow tit	S	
4:43	MJUeta	G	Varied tit	S	
4:43	MJUeta	E	Narcissus flycatcher	S	
4:43	MJUeta	D	Siberian blue robin	S	
4:43	MJUeta	F	Japanese green woodpecker	C	

* Behavior (S: song, C: call, D: drumming)

Supplementary material 4

An example of the presence/absence data of bird vocalization per minute translated from Supplementary material 3.

Vocalization rate (%) is the total minutes a species vocalized divided by the total minutes of all species vocalized multiplied by 100.

Unit time (one minute)	Bird species code											Total
	A	B	C	D	E	F	G	H	I	J	K	
1	1	1	1	1	1	1	1	1	0	0	0	8
2	1	1	1	1	0	1	1	0	1	1	0	8
3	1	1	1	1	1	1	1	0	0	0	1	8
…	…	…	…	…	…	…	…	…	…	…	…	…
Number of unit time with the presence of vocalization	3	3	3	3	2	3	3	1	1	1	1	24
Vocalization rate (%)	12.5	12.5	12.5	12.5	8.3	12.5	12.5	4.2	4.2	4.2	4.2	100
Rank	1	1	1	1	7	1	1	8	8	8	8	

Supplementary material 5 **Bird species detected in the audio and field censuses**

For the audio census, the tabulated abundance index, per species and study year, is the calculated vocalization rate (see Supplementary material 3). The tabulated values for the field census are the sums of the recorded numbers of a species across the four censuses conducted within a respective year. The total time recorded shows the total minutes of all species detected to vocalize, thus giving larger numbers than the total time studied. (-) indicates non-detection. Nomenclature followed the Ornithological Society of Japan, 2012.

		Audio census (%)			Field census		
English name	Scientific name	2011	2012	2013	2011	2012	2013
Copper pheasant	*Syrmaticus soemmerringii*	0.5	0.4	2.0	-	-	1
Oriental turtle dove	*Streptopelia orientalis*	-	0.3	-	-	-	-
Japanese green pigeon	*Treron sieboldii*	3.4	1.7	6.2	2	2	3
Rufous hawk-cuckoo	*Hierococcyx hyperythrus*	1.5	0.1	0.9	1	1	1
Lesser cuckoo	*Cuculus poliocephalus*	0.5	0.3	1.1	-	1	-
Oriental cuckoo	*Cuculus optatus*	3.8	1.6	1.0	2	3	2
Jungle nightjar	*Caprimulgus indicus*	-	0.3	-	-	-	-
Oriental scops owl	*Otus sunia*	-	-	0.2	-	-	-
Japanese pygmy woodpecker	*Dendrocopos kizuki*	0.6	1.1	0.6	1	1	2
White-backed woodpecker	*Dendrocopos leucotos*	-	-	-	-	-	1
Great spotted woodpecker	*Dendrocopos major*	0.02	-	-	2	-	-
Japanese green woodpecker	*Picus awokera*	1.2	1.1	5.6	2	1	2
Eurasian jay	*Garrulus glandarius*	1.5	2.2	2.3	3	2	4
Large-billed crow	*Corvus macrorhynchos*	0.1	0.1	0.1	-	-	1
Goldcrest	*Regulus regulus*	0.7	5.3	-	1	1	-
Willow tit	*Poecile montanus*	1.8	3.0	6.5	3	3	1
Varied tit	*Poecile varius*	3.9	3.3	7.7	4	4	4
Coal tit	*Periparus ater*	28.7	20.5	20.3	8	4	7
Japanese tit	*Parus minor*	1.8	5.8	2.6	4	2	3
Japanese bush warbler	*Cettia diphone*	0.1	0.1	-	-	-	-
Asian stubtail	*Urosphena squameiceps*	-	0.1	-	-	-	-
Long-tailed tit	*Aegithalos caudatus*	-	-	0.3	-	3	-
Japanese leaf warbler	*Phylloscopus xanthodryas*	0.1	-	-	-	1	-
Eastern crowned leaf warbler	*Phylloscopus coronatus*	-	0.4	0.2	-	-	-
Japanese white-eye	*Zosterops japonicus*	0.2	0.2	0.1	-	-	1
Eurasian nuthatch	*Sitta europaea*	5.2	7.7	3.1	3	1	2
Eurasian treecreeper	*Certhia familiaris*	0.6	0.9	1.0	-	-	-
Siberian thrush	*Zoothera sibirica*	1.1	1.7	0.1	-	-	2
Scaly thrush	*Zoothera dauma*	-	0.7	6.2	-	1	-
Brown-headed thrush	*Turdus chrysolaus*	8.9	14.9	10.0	2	3	3
Siberian blue robin	*Luscinia cyane*	7.1	8.9	6.1	4	2	3
Asian brown flycatcher	*Muscicapa dauurica*	-	-	-	-	1	-
Narcissus flycatcher	*Ficedula narcissina*	24.9	16.5	15.6	4	3	3
Blue-and-white flycatcher	*Cyanoptila cyanomelana*	-	0.1	0.1	-	-	1
Eurasian bullfinch	*Pyrrhula pyrrhula*	0.1	-	-	-	-	-
Japanese grosbeak	*Eophona personata*	1.5	0.5	0.1	1	1	-
Meadow bunting	*Emberiza cioides*	-	0.1	-	-	-	-
Red-billed leiothrix	*Leiothrix lutea*	0.04	0.1	0.2	-	-	-
	Total time studied (min)	5295	1890	2310	80	80	80
	Total time recorded (min)	5259	1833	3962	-	-	-
	Total individuals recorded	-	-	-	47	41	47

Supplementary material 6

Identified species numbers in Internet Relay Chat Channel and Twitter hashtag group comments by users listening to the live sound between 5:41 and 6:07 on April 21, 2011 (Japan Standard Time (JST)).

See Figure 5 for a visual representation of this case.

Time (JST)	Internet Relay Chat channel #BirdResearch	Twitter hashtag #tetto	
	Number of different species[1]	Listener code[2]	Twitter comments of hashtag '#tetto'
5:41	3	L3	Drumming sound by a woodpecker knocking on a tree. #tetto
5:42	4		No tweets
5:43	4	L3	Distant and short sounds, Fi..fi.. What's that? Continued drumming at 5:52. #tetto
5:44	2		No tweets
5:45	3	L4	Is it a white-backed woodpecker? #tetto
		L1	I heard a Eurasian nuthatch, but the louder sound was a sika deer. RT L3:5:42
5:46~50	4		No tweets
5:51	3		No tweets

5:52	3	L3	If it was a deer, it's the alert sound. I have heard a recording of it before. RT L1: I heard a Eurasian nuthatch, but the louder sound was sika deer. #tetto
5:53	3	L4	Ke! Ke! It might be a Japanese green woodpecker. #tetto
5:54~58	3		No tweets
5:59	3	L1	The deer is on alert for what? A bear? There is no sound of bear movement, but it's their time to emerge from hibernation soon. #tetto
6:00	3		No tweets
6:01~02	4		No tweets
6:02	4		No tweets
6:03	3	L3	I feel an expanse of space by hearing the voice of the bird which is flying while crying. It sounds like several woodpeckers are drumming together. #tetto
6:04~06	3		No tweets
6:07	4	L5	This live sound from Oku-Chichibu forest is extremely rich in bird diversity. Looking at those comments, I feel I was taken on a private tour of a museum lead by ornithologists. I appreciate that. #tetto

[1] Number of species identified by the ornithologist surveyors per minute with live sound; Listener L1 determined the eventual species name.

[2] L1-L3 were authors, L4 an ornithologist using Twitter, and L5 someone listening in.
